# The Adult, Larva, and Pupa of a New *Pseudopyrochroa* (Coleoptera: Pyrochroidae: Pyrochroinae) from China, with Molecular Phylogenetic Inferences †

**DOI:** 10.3390/insects12121089

**Published:** 2021-12-04

**Authors:** Zhao Pan, Jia-Chong Duan, Qi Gao, Daniel K. Young

**Affiliations:** 1 Key Laboratory of Zoological Systematics and Application of Hebei Province, College of Life Sciences, Institute of Life Science and Green Development, Hebei University, Baoding 071002, China; panzhao86@yeah.net (Z.P.); duanjiachong@126.com (J.-C.D.); gao_qii@126.com (Q.G.); 2 Department of Entomology, University of Wisconsin, Madison, WI 53706, USA

**Keywords:** *Pseudopyrochroa reni*, new species, morphological description, *COI*, Mt. Qinling

## Abstract

**Simple Summary:**

Mt. Qinling is one of the priority areas for biodiversity conservation in China. However, far less is currently known about the species richness, abundance, natural history, and phenology of insects from the mountains, especially from the western region. During the investigation of insect biodiversity from the western region of Mt. Qinling, the first species belonging to the genus *Pseudopyrochroa* of the family Pyrochroidae was discovered and is described herein as *Pseudopyrochroa reni* Pan & Young, **n. sp**. The larva and pupa of this new species were associated with the adults using mtDNA *COI* fragment sequences. Below, the larva, pupa, and adult male and female are described and illustrated. A very preliminary analysis of phylogenetic relationships among five genera and 14 species of Pyrochroidae is provided and discussed based on *COI* sequences. Lastly, the fauna of Pyrochroidae from the Mt. Qinling biodiversity conservation area is briefly discussed.

**Abstract:**

A new species of *Pseudopyrochroa* Pic, 1906, *P. reni* Pan & Young, **n. sp.**, is described from the western region of Mt. Qinling, China. Larvae, pupae, and adults were associated using molecular phylogenetic analyses based on mtDNA *COI* barcode sequences. All three stages are described and illustrated. Additionally, preliminary phylogenetic relationships among five genera and 14 species of Pyrochroidae, including *Pseudopyrochroa*, are hypothesized based on *COI* sequence data. The fauna of Pyrochroidae from the Mt. Qinling biodiversity conservation area is discussed.

## 1. Introduction

The fire-colored beetle genus *Pseudopyrochroa* Pic, 1906 is the most speciose of the family Pyrochroidae Latreille, 1807, with approximately 70 species, distributed in the eastern Palearctic and the Oriental Regions [1,2,3]. Some species of the genus were taxonomically studied by Blair [4], Kôno [5], and Kai and Yoshitomi [2]. Adults are distinguished from other Asian pyrochroine genera by the combination of the following characters: compound eyes small, separated (♂) by a space at least as wide as eye width; dorsal outline of head ovate, genae not prominent in posterolateral aspect; paired, interocular cranial pits (♂) usually well developed; parameres narrowly separated distally, their dorsolateral apices bifurcately toothed, with each tooth projecting basally; apex of penis provided with a dorsomesal, basally recurved hook [1,4,6,7,8]. The paucity of material and species associations has precluded diagnoses for larvae and pupae of *Pseudopyrochroa*.

Although exhibiting robust species richness, *Pseudopyrochroa* is poorly known from China. Currently, 20 species of *Pseudopyrochroa* have been known from China [3,8]; largely described by Young (eight species) [7,8,9,10] and Pic (seven species) [11,12,13,14,15]. Among them, larvae of three species from Taiwan were described [16,17] and the larva of *P. lateraria* (Motschulsky, 1860) was included in a key to the larvae of Japanese pyrochroid species by Yoshitomi and Kai [18]. No pupae of *Pseudopyrochroa* have previously been described.

During the summer of 2020, Prof. Guo-Dong Ren, a colleague of the author (Z.P.), and several of his students collected adults, larvae, and pupae of Pyrochroidae from the western region of Mt. Qinling that is located in eastern Gansu province and western Shaanxi province, China. After comparing the adults with known *Pseudopyrochroa* species, we concluded that there is one new species in this material. The larva and pupa of this new species were associated using mtDNA *COI* fragment sequences (see below). Below, we describe the larva, pupa, and adult male and female of the new species of *Pseudopyrochroa*. We also provide its *COI* sequences and use these molecular data as the basis for a very preliminary examination of phylogenetic relationships within Pyrochroidae.

## 2. Material and Methods

### 2.1. Morphological Examination

Fifteen adults, 52 larvae, and 16 pupae were examined for this study and have been deposited at the Museum of Hebei University, Baoding, China (MHBU) and personal collection of Daniel K. Young (DYCC).

Anatomical terms used in the descriptions were introduced by Young [19] and have been used elsewhere [8,16,17].

The preparation of larvae follows Young et al. [16]. The figures were taken with three distinct imaging systems: (a) Canon EOS 5D Mark III (Canon Inc., Tokyo, Japan) connected to a Laowa FF 100 mm F2.8 CA-Dreamer Macro 2× or Laowa FF 25 mm F2.8 Ultra Macro 2.5–5× (Anhui Changgeng Optics Technology Co., Ltd, Hefei, China); (b) Leica M205A stereomicroscope equipped with a Leica DFC450 camera (Leica Microsystems, Singapore, Singapore), which was controlled using the Leica application suite 4.3; (c) JVC^®^ KY-F75U (JVC Kenwood, Long Beach, CA, USA) digital camera attached to a Leica Z16 APO dissecting microscope (Leica Microsystems, Buffalo Grove, IL, USA) with an apochromatic zoom objective and motor focus drive, using a Syncroscopy^®^ Auto-Montage System (Synoptics, Cambridge, UK) and software. Multiple images were used to construct the final figures. Images were illuminated with either an LED ring light attached to the end of the microscope column, with incidental light filtered to reduce glare, or by a gooseneck illuminator with bifurcating fiberoptics; image stacks were white-balance-corrected using the system software (Synoptics, Cambridge, UK). Montaged images were edited using Adobe Photoshop^®^ v22.1.0 to form the final figure plates.

Label data are presented verbatim. Line breaks on labels are denoted by a double slash (//); metadata and notes (not written on the labels, themselves) are presented in square brackets ([]). Scientific names are uniformly presented in italics.

### 2.2. Molecular Analyses

As noted above, during the summer of 2020, 52 pyrochroid larvae and 16 pupae were collected in the field together with adults. In an attempt to associate the different stages, eight individuals (one male adult, one female adult, three larvae, and three pupae) were selected and molecularly analyzed. A preanalysis indicated at least three morphospecies in our samples. To resolve species identity, 29 *COI* barcode sequences associated with 11 species level identifications from four genera of Pyrochroidae were selected and downloaded from GenBank. Additionally, one *COI* sequence of *Boros schneideri* (Panzer, 1796) of the family Boridae Thomson, 1859 was selected. Boridae has been consistently recovered as a near relative of Pyrochroidae [20,21,22,23,24] and was chosen as the outgroup. The details relating to these samples are provided in Table 1.

For our samples, total genomic DNA was extracted from the leg muscles using the Insect gDNA Isolation Kit (Biomiga, Hangzhou, China). Fragments of the mitochondrial molecular marker *COI* for DNA barcoding were amplified with the primer pair LCO1490 and HCO2198 [29]. Polymerase chain reaction (PCR) amplifications were performed with the settings used in Liu et al. [30]. The PCR products were examined using 1.0% agarose gel electrophoresis and purified and sequenced by General Biol (Chuzhou, China).

DNA sequences were edited in DNASTAR SeqMan Pro v.7.1.0 (DNASTAR, Inc., Madison, WI, USA) and aligned using the MAFFT online service [31]. All newly generated sequences were deposited in GenBank (Table 1).

Genetic distances were calculated by using the Kimura 2-parameter model with MEGA v10.2.6 [32]. Phylogenetic analyses were performed under the assumptions of maximum likelihood (ML) [33]. A best-fit model was tested according to the corrected Akaike’s Information Criterion (AICc) using ModelFinder (included in IQ-TREE) [34] with the software PhyloSuite v1.2.2 [35]. The ML tree search was performed in IQ-TREE v1.6.8 [36] that was also plugged into PhyloSuite. Clade support was assessed using ultrafast bootstrap values.

## 3. Results

***Pseudopyrochroa reni* Pan & Young, n. sp.**

www.zoobank.org/urn:lsid:zoobank.org:pub:7FE0DEBE-9850-4467-850C-897888610C50, accessed on 8 November 2021.

**Type locality.** China, Gansu Province, Tianshui City, Maiji District, Liyuan Forestry Farm.

**Type specimens (Adults).** Holotype: ♂, with the following labels: “2020.VIII.1//Gansu, Maiji, Liyuan Forestry Farm//Guo-Dong Ren et al.//Museum of Hebei University” [in Chinese], “34°26.782′ N//106°31.256′ E//Elev. 1065 m//Museum of Hebei University”, “P1B10”, “HOLOTYPE//*Pseudopyrochroa reni* **n. sp.**// Det. Pan & Young” (MHBU).

Paratypes (6♂♂ and 9♀♀ in total): 6♂♂ and 2♀♀, idem., “P1B4–P1B9/P1C1/P1C2” (2♂♂ and 1♀ DYCC, others MHBU); 1♀, “2020.VIII.1//Gansu, Maiji, Longmen Forestry Farm//Guo-Dong Ren et al.//Museum of Hebei University” [in Chinese], “34°12.607′ N//106°24.032′ E//Elev. 1347 m//Museum of Hebei University”, “P1A6” (MHBU); 2♀♀, “2020.VII.30//Gansu, Maiji, Liyuan Forestry Farm//Guo-Dong Ren et al.//Museum of Hebei University” [in Chinese], “34°24.772′ N//106°29.128′ E//Elev. 1328 m//Museum of Hebei University”, “P1B1/P1B2” (MHBU); 3♀♀, “2020.VIII.3//Gansu, Maiji, Tailu Forestry Farm//Guo-Dong Ren et al.//Museum of Hebei University” [in Chinese], “34°27.489′ N//106°13.014′ E//Elev. 1451 m//Museum of Hebei University”, “P1D6–P1D8” (MHBU); 1♀, “Date: 1992.VIII.7//Locality: Gansu, Longnan City, Hui County, Laoyegou//Jinqian Liu leg.” [in Chinese] (MHBU). All paratypes have the label “PARATYPE//*Pseudopyrochroa reni* **n. sp.**// Det. Pan & Young”.

**Other examined materials. Larvae.** 1 ex., “2020.VII.30//Gansu, Maiji, Liyuan Forestry Farm//Guo-Dong Ren et al.//Museum of Hebei University” [in Chinese], “34°24.772′ N//106°29.128′ E//Elev. 1328 m//Museum of Hebei University]”, “P1B3” (MHBU); 19 exx., “2020.VIII.1//Gansu, Maiji, Liyuan Forestry Farm//Guo-Dong Ren et al.//Museum of Hebei University” [in Chinese], “34°26.782′ N//106°31.256′ E//Elev. 1065 m//Museum of Hebei University”, “P1C3–P1C10” (MHBU); 6 exx., “2020.VIII.1//Gansu, Maiji, Liyuan Forestry Farm//Guo-Dong Ren et al.//Museum of Hebei University” [in Chinese], “34°27.254′ N//106°29.052′ E//Elev. 1359 m//Museum of Hebei University”, “P1D3/P1D4” (MHBU); 4 exx., “2020.VIII.3//Gansu, Maiji, Tailu Forestry Farm//Guo-Dong Ren et al.//Museum of Hebei University” [in Chinese], “34°27.489′ N//106°13.014′ E//Elev. 1451 m//Museum of Hebei University”, “P1E10” (1 DYCC, 3 MHBU); 2 exx., “2020.VIII.3//Gansu, Maiji, Tailu Forestry Farm//Guo-Dong Ren et al.//Museum of Hebei University” [in Chinese], “34°30.641′ N//106°13.248′ E//Elev. 1138 m//Museum of Hebei University”, “P1G1/P1G2” (MHBU).

**Pupae.** 1♂, “2020.VIII.1//Gansu, Maiji, Liyuan Forestry Farm//Guo-Dong Ren et al.//Museum of Hebei University” [in Chinese], “34°26.782′ N//106°31.256′ E//Elev. 1065 m//Museum of Hebei University”, “P1D1” (MHBU); 3♂♂ and 3♀♀, “2020.VIII.3//Gansu, Maiji, Tailu Forestry Farm//Guo-Dong Ren et al.//Museum of Hebei University” [in Chinese], “34°27.489′ N//106°13.014′ E//Elev. 1451 m//Museum of Hebei University”, “P1D9/P1D10/P1E1–P1E4” (MHBU); 1♂ and 8♀♀, “2020.VIII.3//Gansu, Maiji, Tailu Forestry Farm//Guo-Dong Ren et al.//Museum of Hebei University” [in Chinese], “34°30.641′ N//106°13.248′ E//Elev. 1138 m//Museum of Hebei University”, “P1F1–P1F6” (1 pair DYCC, others MHBU).

**Figure 1:**
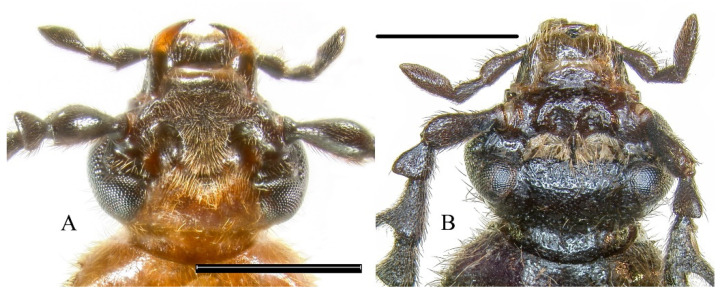
Head of *Pseudopyrochroa* spp., dorsal view. (**A**) *P. punctifrons* Young; (**B**) *P. lateraria* (Motschulsky). Scale bars: 1 mm.

**Figure 2:**
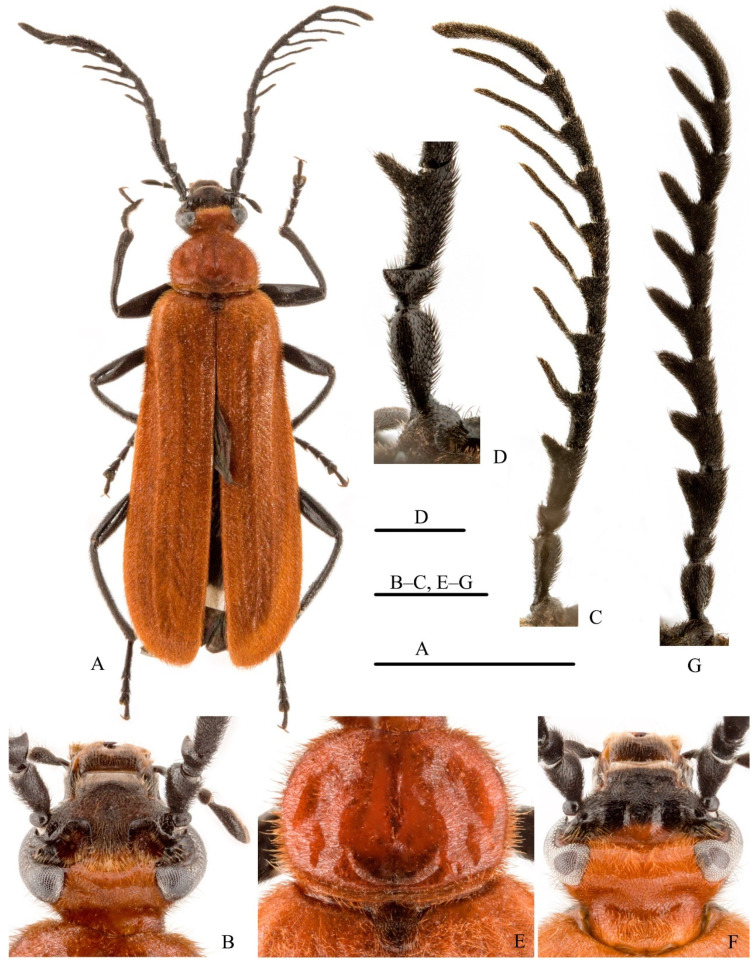
Adult of *Pseudopyrochroa reni* Pan & Young, **n. sp.**, paratypes. (**A**) Habitus, male, dorsal view; (**B**) Head, male, dorsal view; (**C**) Antenna, male; (**D**) Antennomeres I–III, male; (**E**) Pronotum and scutellar shield, male; (**F**) Head, female, dorsal view; (**G**) Antenna, female. Scale bars: 5 mm (**A**), 1 mm (**B**,**C**, **E**–**G**), 0.5 mm (**D**).

**Diagnosis. Adult.** The new species can be easily distinguished from most other *Pseudopyrochroa* species by the position of the male cranial pits. A possible exception is *P. punctifrons* Young, 2000 from Taiwan Island. *Pseudopyrochroa punctifrons* and *P. reni* **n. sp.** are also similar in body coloration. The cranial pits are subreniform in *P. punctifrons* (Figure 1A), but more rounded in *P. reni* **n. sp.** (Figure 2B); the extension of frons between eyes is almost flat in *P. punctifrons*, but slightly depressed in *P. reni* **n. sp.**; the outer apical angle of the antennal pedicel of *P. reni* **n. sp.** is more stronger produced than that of *P. punctifrons* (Figure 1A and Figure 2C); the terminal maxillary palpomere is more elongate in *P. reni* **n. sp.**, approximately 3.5× as long as wide (Figure 2B), and only 2.6× as long as wide in males of *P. punctifrons* (Figure 1A). Males of *Pseudopyrochroa lateraria* (Motschulsky, 1860) from the Far East of Russia and northeastern China have a similarly shaped antennal pedicle to that of *P. reni* **n. sp.**, but may be easily distinguished in the coloration of the head and the position of the cranial pits (Figure 1B).

**Larva.** Anatomically, the mature larva of *P. reni* **n. sp.** conforms to the preliminary diagnosis for larvae of *Pseudopyrochroa* [16,17,37]. The larva of *P. reni* **n. sp.** differs from those of other congeners, e.g., *P. carinifrons* Kôno, 1929, *P. depressa* Pic, 1914, and *P. fainanensis* (Pic, 1911) (all distribute in Taiwan, China), by the structure of the urogomphal plate. Mature larvae of *P. reni* have the lateral lobes of urogomphal plate rounded; the urogomphi are straight and appear slightly divergent, not curved at apex; the urogomphal lip is distinctly visible in dorsal view and widely rounded; the urogomphal pits are deep and without rugulae.

**Pupa.** This is the first pupa of *Pseudopyrochroa* to be described. Compared with pupae of related pyrochroid genera (e.g., *Dendroides* Latreille, 1810, *Schizotus* Newman, 1838, *Neopyrochroa* Blair, 1914 [19]), the pupa of *P. reni* **n. sp.** may be distinguished by the following characters: abdomen with two lateral marginal tubercles, one simple tubercle anteriad and one large, bifurcate tubercle posteriad (*Dendroides* spp. and *Schizotus cervicalis* Newman, 1838 possess three lateral marginal tubercles); setae apical on all tubercles (setae on tubercles arising preapically in *Neopyrochroa* spp.); posterior marginal tubercles greatly narrowed distally from the mid-length (but slightly narrowed in the pupa of *S. cervicalis*).

**Descriptions. Adult.***Male.* Length 10.9–14.8 mm, humeral width 2.8–3.4 mm; maximal elytral width 3.8–4.6 mm (n = 6). Basal half of head, pronotum, scutellar shield, and elytra generally orange to red; anterior margin of labrum and apical half of mandibles orange-brown; otherwise black (Figure 2A,B).

Head: Dorsal cranial surface generally finely, densely punctate, moderately setose; frons coarsely, densely punctate. Interocular cranial pits (Figure 2B) paired, deeply and distinctly impressed, each with inwardly decumbent golden setae; positioned very close to compound eyes and antennae, and completely separated mesally by broad, densely setose posteriomesal extension of frons. Vertex broadly convex, anterior face beset with anteriorly decumbent setae that serve to clothe posteriomesal rims of cranial pits. Antennae (Figure 2C) with scape and pedicel shining, shallowly, moderately densely punctate, moderately clothed with short, black setae; scape elongate, somewhat enlarged distally, subconical, approximately twice as long as wide, and 2.6× length of pedicel (Figure 2D); antennal pedicel short, broadly triangulate, approximately 0.45× length of flagellomere I, with outer apical angle distinctly, acutely produced (Figure 2D); flagellum densely setose, strongly, delicately pectinate, rami of flagellomeres with gradually increasing lengths, ramus of flagellomere I short, distinctly shorter than II, ramus of II slightly shorter than III, ramus of III as long as IV, rami of IV–VII longer than preceding flagellomere, ramus of VIII shorter than flagellomere IX (Figure 2C).

Thorax: Pronotum (Figure 2E) transversely ovate in dorsal view, 1.3× as wide as long; shining, very shallowly and sparsely punctulate, vestiture consisting of a moderately dense covering of fine erect yellowish to golden setae; disc with two depressions on each side, one subrounded depression in middle of base, one longitudinal, mesal furrow at center, and one transverse furrow along basal margin; marginal bead well developed. Scutellar shield densely covered with retrorsely decumbent, yellowish to golden, moderately coarse setae. Elytra elongate, covering abdomen, distinctly wider apically, longitudinal costae obsolete; elytral vestiture consisting of short, dense, erect to decumbent yellowish-golden setae.

**Figure 3:**
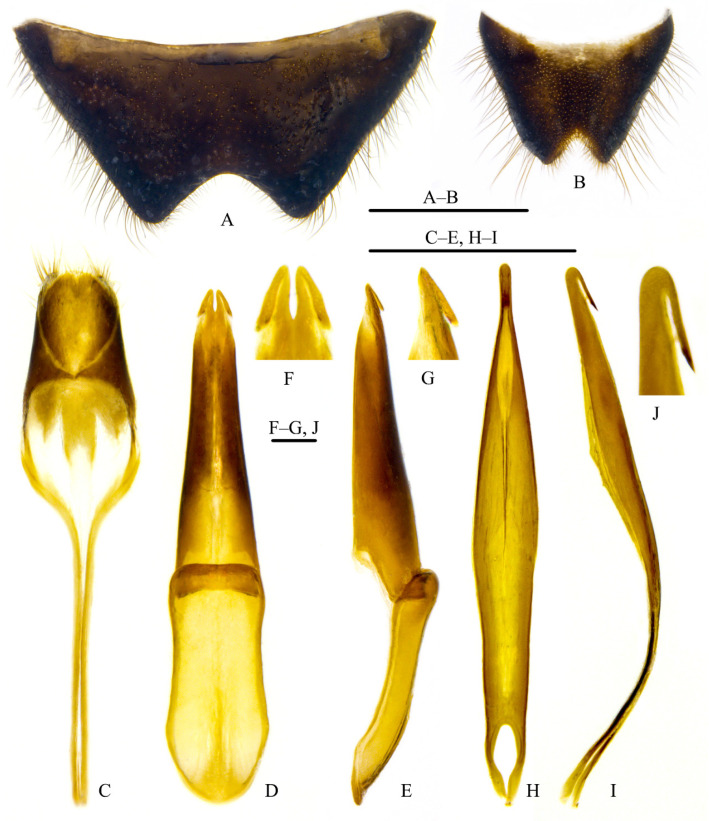
Adult of *Pseudopyrochroa reni* Pan & Young, **n. sp.**, paratype, male. (**A**) Sternite VII, ventral view; (**B**) Sternite VIII, ventral view; (**C**)Tergite and sternite IX–X, dorsal view; (**D**,**E**) Tegmen: (**D**) dorsal view, (**E**) lateral view; (**F**,**G**) Apex of paramere: (**F**) dorsal view, (**G**) lateral view; (**H**,**I**) Penis: (**H**) dorsal view, (**I**) lateral view; (**J**) Apex of penis, lateral view. Scale bars: 0.1 mm (**F**,**G**,**J**), 1 mm (others).

Abdomen: Sternite VII narrowed apically, its distal margin obtusely emarginate and depressed at center along margin (Figure 3A); sternite VIII narrowed apically, its distal margin deeply, acutely emarginate (Figure 3B); sternite and tergite IX–X as in Figure 3C. Genitalia as in Figure 3D–J: parameres largely fused, narrowly separated apically, each bearing a recurved hook (Figure 3E–G); penis elongate, somewhat dorsoventrally flattened, tapered apically and produced into a bluntly recurved hook (Figure 3H–J).

*Female.* Length 12.2–16.7 mm, humeral width 3.0–4.1 mm; maximal elytral width 4.1–5.1 mm (n = 9). Slightly larger than male, lacking cranial pits, but with interocular region shallowly, transversely sulcate between emarginations of compound eyes (Figure 2F). Antennae with all segments, including scape and pedicel, densely setose, velvet-like in appearance; pedicel small, outer angle not produced (Figure 2G); flagellum pectinate, flagellomeres larger, more robust than those of male; ramus of each flagellomere distinctly shorter than those of male, shorter than or as long as that of preceding flagellomere. Posterior margin of sternite VII straight, not emarginate; sternite VIII largely membranous.

**Figure 4:**
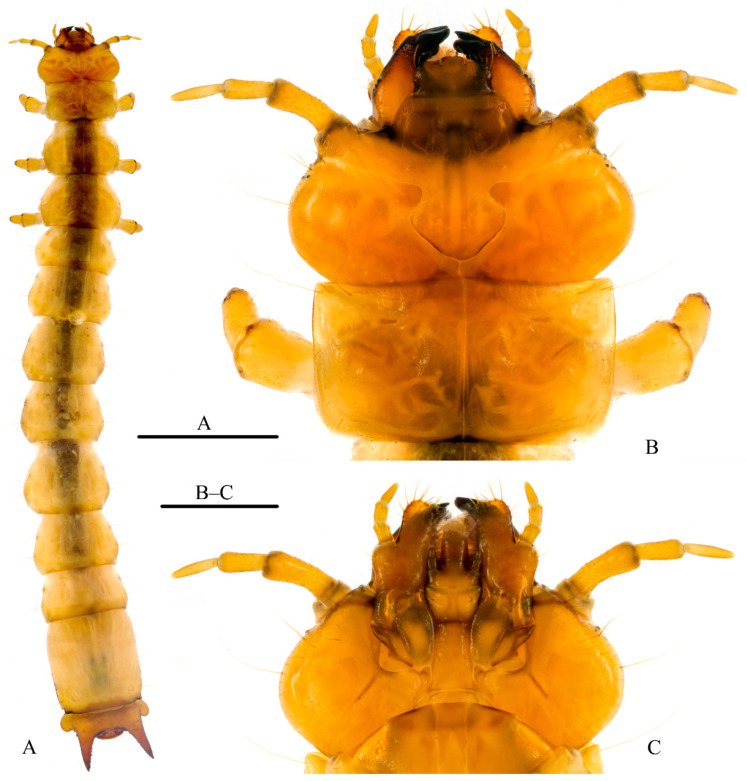
Larva of *Pseudopyrochroa reni* Pan & Young, **n. sp.** (**A**) Habitus, dorsal view; (**B**) Head and pronotum, dorsal view; (**C**) Head, ventral view. Scale bars: 5 mm (**A**), 1 mm (**B**,**C**).

**Larva.** Mature larvae (Figure 4A; n = 13) attain lengths (mesal labral apex to apex of urogomphal lip) of 23.1–28.3 mm and widths (across widest portion of abdominal segment VIII) of 3.2–4.0 mm. Body (Figure 4A) orthosomatic with sides subparallel, moderately sclerotized except much of cranium, mandibles, and urogomphal plate more heavily sclerotized; body vestiture consisting of short to moderately elongate, scattered setae. Thoracic and abdominal terga lacking parabasal ridges. Head and body creamy-yellowish to amber, melanization much darker in areas of heavy sclerotization such as tips of mandibles, urogomphi, urogomphal lip, and urogomphal pits.

Head (Figure 4B,C): Prognathous, flattened, exserted from prothorax. Epicranial suture (Figure 4B) lyriform with stem short, frontal arms complete nearly to antennal insertions; endocarinae absent. Free, symmetrical labrum anteriad fused frontoclypeal region. Four stemmata on each side, in 2 groups of 2, lower pair parallel with antennal insertions. Antennal insertions fully exposed, antennae (Figure 4B,C) moderately elongate, filiform, 3-segmented, antennomere I 0.2–0.3 longer than antennomeres II and III; sensorium of II small, conical; III narrower than antennomeres I–II, acutely rounded apically. Mouthparts retracted. Mandibles (Figure 4B) heavily sclerotized, asymmetrical, molar area of each mandible well developed, left mandible bearing a prominent molar tooth; apex of right mandible distinctly tridentate, that of left mandible bidentate; dorsal mandibular surface basad molae with microtrichial complex well developed. Maxillae (Figure 4C) each with 1-segmented cardo diagonally folded upward upon itself toward stipes and thus appearing 2-segmented, and a well-developed, undivided, pad-like maxillary articulating area; ventral surface of stipes with dense row of stout setae mesad palpifer along adoral margin; galea and lacinia fused to form maxillary mala; mala bearing stout apical and adoral setae and spinose-dentiform uncus at apico-adoral margin; each maxillary palpus 3-segmented, filiform, palpomeres I–III subequal in length, III tapering distally, acutely rounded apically. Labium (Figure 4C) with mentum ovate-subquadrate, submentum elongate with sides shallowly sinuate basally, apical margin slightly more heavily sclerotized, convexly rounded; ligula well developed, elongate; each labial palpus short, 2-segmented, palpomere II about half of I in length. Hypopharyngeal sclerome well sclerotized, molar-like; proximal region of hypopharynx with complex setal brushes. Hypostomal rods (Figure 4C) well developed, divergent; gular sutures separate.

Thorax: Thoracic segmentation well developed, sides parallel; pronotum (Figure 4B) transversely subrectangular, sides parallel; mesonotum and metanotum transversely sub-ovate, widened posteriorly; cervicosternum divided into three plates. Legs well developed, moderately short, 5-segmented including tarsungulus, vestiture consisting of sparse, short setae; coxae large, separated by 2–3 coxal diameters.

**Figure 5:**
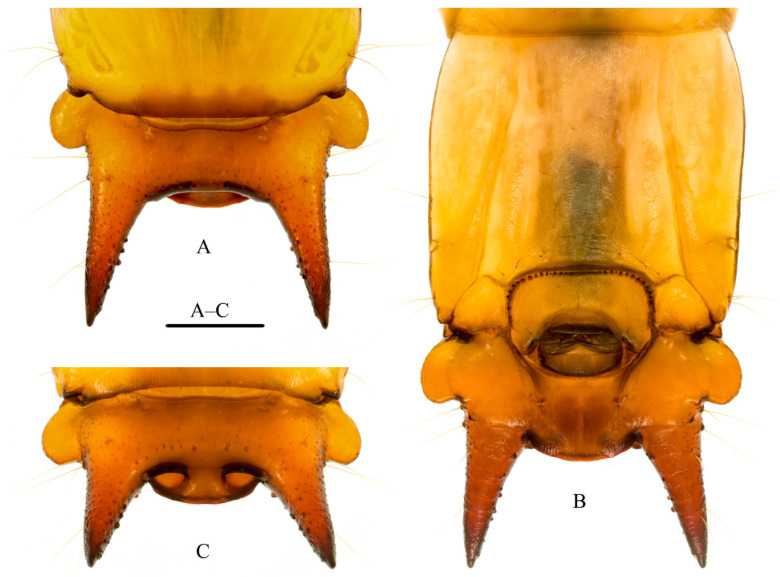
Larva of *Pseudopyrochroa reni* Pan & Young, **n. sp.** (**A**) Urogomphal plate and urogomphi, dorsal view; (**B**) Sternite VIII–X, urogomphal plate, and urogomphi, ventral view; (**C**) urogomphal pits and urogomphal lip, posterior view. Scale bar: 1 mm.

Abdomen: Flattened, moderately sclerotized, tergites I–VII subequal in length and width; VIII longitudinally subrectangular, approximately combined length of VI + VII, sides parallel. Sternite VIII (Figure 5B) emarginate apically. Tergite IX forming urogomphal plate (Figure 5A–C), widest basally where it forms well developed rounded lateral lobes; surface of urogomphal plate bearing numerous, well-developed callosities and several larger, dorsal and lateral setigerous calli on dorsal and lateral surfaces of urogomphi; urogomphi heavily sclerotized, moderately long, straight, slightly divergent, tapering and acuminate apically; ventral surface of urogomphal plate shallowly, but sharply excavate basally at articulation with sternites IX and X, excavation narrowing distally to bases of urogomphi and urogomphal lip. Urogomphal plate possessing a heavily sclerotized, mesally rounded, produced urogomphal lip ventrally, between two, heavily sclerotized, deep urogomphal pits (Figure 5C), which, in turn, arise distally between heavily sclerotized, fixed urogomphi; urogomphal pits smooth, without any apparent rugulae (Figure 5C). Sternite IX (Figure 5B) broadly, transversely U-shaped, divided mesally by weak, desclerotized line, partially recessed into shallow emargination of sternite VIII, possessing continuous semicircular arch of approximately 30–40 well-developed asperities along anterior margin. Segment X (Figure 5B) reduced, transversely ovate, basal margin rounded, recessed into emarginations of sternite IX, visible ventrally.

Spiracles: One pair of well-developed, ovate thoracic spiracles, situated ventrolaterally on laterotergite along anterior end of mesothorax. Paired, ovate abdominal spiracles, subequal in size, located on dorsolateral margin of abdominal tergite I and ventrolateral margins of abdominal laterotergites II–VII; paired spiracles of abdominal laterotergite VIII (Figure 5B) located ventrolaterally at distal 2/7 (0.29) of its length.

**Figure 6:**
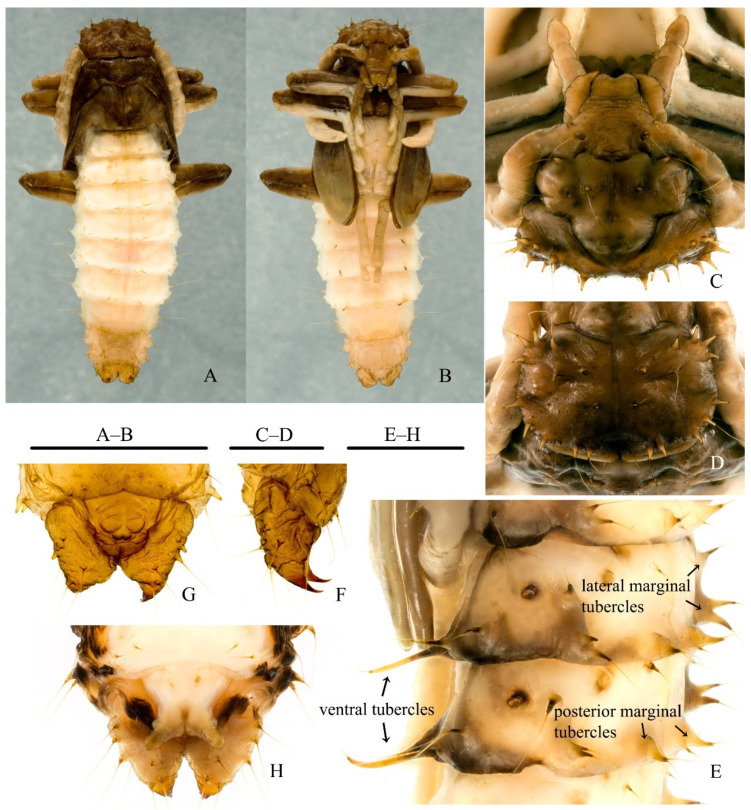
Pupa of *Pseudopyrochroa reni* Pan & Young, **n. sp.** (**A**,**B**) Habitus, male: (**A**) dorsal view, (**B**) ventral view; (**C**) Head, male; (**D**) Pronotum, male; (**E**) Abdominal segments III–IV, female, dorsolateral view; (**F**) Urogomphi, male, lateral view; (**G**) Sternite IX and male genitalia, ventral view; (**H**) Sternite IX and female ovipositor, ventral view. Scale bars: 5 mm (**A**,**B**), 1 mm (**C**–**H**).

**Pupa.***Male.* Length (anterior margin of pronotum to apices of urogomphi) 9.8–11.7 mm and widths (across widest portion of abdominal segment IV) 3.0–3.2 mm (n = 4). Body (Figure 6A,B) moderately flattened dorsoventrally, sides of abdomen subparallel; moderately sclerotized; body vestiture consisting of several tubercles, most of which bear long apical setae. Body creamy-yellowish to brown, usually melanization much darker on head, pronotum, legs, wings, urogomphi, and posterior and lateral margins of pterothoracic terga and abdominal segments I–VIII; darker areas expanded in more mature individuals.

Head (Figure 6C): Deflexed ventrally, not visible in dorsal view. Dorsal cranial surface smooth, with 12 tubercles: 4 transversely arranged on vertex, 1 on inside of each eye, and 3 on inside of each antennal insertion (1 anteriad and 2 posteriad). Frons with 2 deep depressions positioned corresponding to interocular cranial pits of male adult, and 1 transverse furrow connecting above 2 depressions. Mouthparts and antennae incompletely segmented.

Thorax: Pronotum (Figure 6D) subtrapezoidal, with moderately curved lateral and anterior margins; on each side of disc, divided by ecdysial line, with 4 tubercles positioned at each anterior angle, 2 tubercles along each subapical line, 1 submesally on either side of ecdysial line, 3 tubercles along posterior half of each lateral margin, and 2 tubercles subposteriad on each side of meson; disc with 2 large, mesal depressions, one on either side of ecdysial line, and 1 shallow, small, mesal depression basally. Mesonotum shorter than pro- and metanotum, with 2 tubercles arranged diagonally on posterior 1/4 of each side. Metanotum longer than mesonotum, but shorter than pronotum, disc also with 2 tubercles each, placed similarly to those on mesonotum. Elytra and metathoracic wings clearly developing, extending backwardly to posterior margin of abdominal sternite III. Legs incompletely segmented; each femur with 3 large tubercles in ventral view: 2 subapically and 1 at apical 1/4 of each ventral margin.

Abdomen: Subfusiform, widest at segment IV; segments I–VII moderately sclerotized, subequal in length, posterior margins of tergites I–VI and sternites I–VII almost straight, and that of tergite VII widely rounded; segments VIII and IX more membranous. Segments I–VII smooth, with almost same tubercle pattern (as in Figure 6E): tergites with 2 lateral marginal tubercles (1 simple tubercle anteriad and 1 large, bifurcate tubercle posteriad, but tergite I without anterior tubercle), 4 posterior marginal tubercles, in 2 groups of 2 (inner one very small, subuliform, and outer one large, greatly narrowed at mid-length), and 2 small anterior marginal tubercles (invisible on I and VII in some cases); laterotergites each with 1 pleural tubercle; sternites III–VI with 4 ventral tubercles, in 2 groups of 2, on subposterior margin (inner one elongate, slender, and curved; outer one distinctly shorter, approximately half length of inner tubercule); ventral tubercles absent on sternites I and II, and minute on sternite VII. Segment VIII wrinkled, with 2 posterior marginal tubercles, 3 lateral marginal tubercles similar to those on I–VII, 1 min pleural tubercle, and 4 min ventral tubercles. Segment IX also wrinkled, with 12 tubercles; urogomphi (Figure 6F) heavily sclerotized, short, pointed apically, and curved dorsally. Segment X and genitalia as in Figure 6G.

Spiracles: Abdominal spiracles (Figure 6E) located on dorsolateral margin of abdominal tergites I-VI, paired, ovate, larger on I than that on II-VI.

*Female*. Length 10.3–13.4 mm, abdominal width 3.1–4.1 mm (n = 10). Depressions on head distinctly shallower than those in male. Posterior margin of tergite VII rounded in middle, and that of sternite VII widely rounded. Ovipositor as in Figure 6H.

**Molecular analyses.** Thirty-eight mitochondrial *COI* sequences were obtained, including those downloaded from GenBank, which were pruned to lengths of 657 bp after sequence alignment.

**Figure 7:**
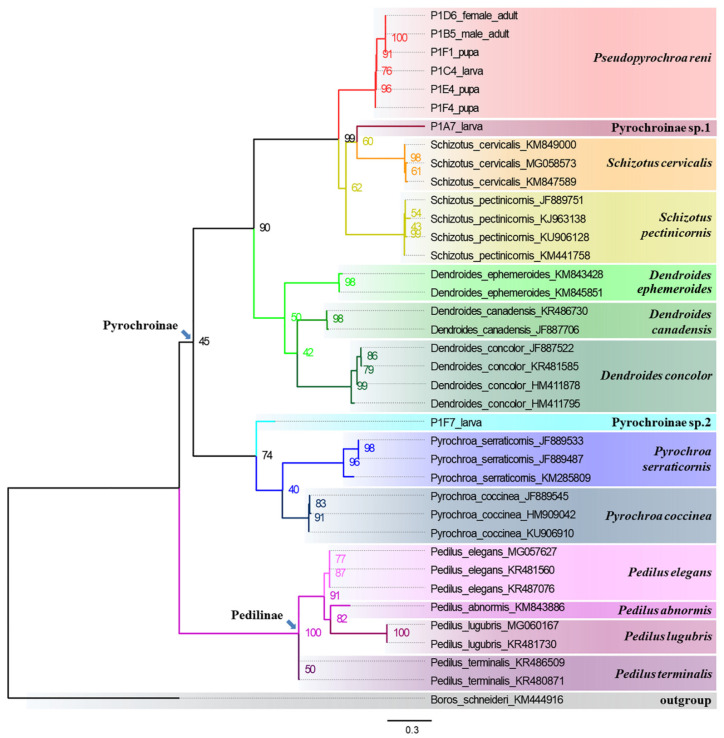
Maximum likelihood phylogenetic tree based on *COI* sequence data. Support for each node is represented by ultrafast bootstrap values (uBV, %).

Maximum likelihood phylogenetic analysis was inferred under the MGK + G4 model for 5000 ultrafast bootstraps [38], as well as the Shimodaira–Hasegawa–like approximate likelihood-ratio test [39]. The ML tree (Figure 7; Log-likelihood= −3284.871) revealed that larval sample P1C4 and the pupal samples P1E4, P1F1, and P1F4 were clustered with the adults of *P. reni* **n. sp.** (P1B5 and P1D6) in a single clade (uBV = 96%). Meanwhile, larval samples P1A7 and P1F7 fell outside the *P. reni* **n. sp.** cluster, with P1A7 relatively close to *Schizotus cervicalis*, although with low uBV (60%). The P1F7 larva formed a sister group with the clade of two *Pyrochroa* species (uBV = 74%). Based on the above results, samples P1C4, P1E4, P1F1, and P1F4 are hypothesized to be conspecific with *P. reni* **n. sp.** adults and were tentatively placed into one group for the following genetic distances analyses.

The maximum and mean intraspecific genetic distances for each species are listed in Table 2. The interspecific mean distances ranged from 4.64% to 18.86%. The results indicate that the maximum genetic distances of the tentative *P. reni* **n. sp.** cluster are evidently lower than the minimum interspecific divergence (2.17% < 4.64%). Therefore, we conclude that the above assumption is correct and samples P1C4 and P1E4, P1F1, P1F4 are the larva and pupae of *P. reni* **n. sp.**, respectively.

**Etymology.** After the specific epithet is a patronym to honor Prof. Guo-Dong Ren, Hebei University, who collected and supported us with almost all specimens of this new species.

**Distribution.** China: Gansu (Longnan, Tianshui).

## 4. Discussion

### 4.1. Taxonomic Remarks

The larvae were collected in the field; hence, it was not possible to judge the instar. We measured all larvae, and we determined that individuals with a maximum width ≥3.0 mm are mature. The larval description above was based on these, presumably final instar larvae.

With the larvae of only a relatively small number of species described thus far, our understanding of the anatomical diversity within *Pseudopyrochroa* is clearly in its infancy. Thus, it would be premature to attempt a diagnosis for larvae of the genus. Rather, it should be stressed that larvae of Pyrochroidae generally have been found to offer numerous characters for species recognition. In light of this observation, we hope to continue to discover additional larvae and associate them with their respective adults by rearing and/or *COI* sequence data. Only with the discovery of larvae for additional species of *Pseudopyrochroa*, as well as the several other pyrochroid genera for which we have no larval associations, can we begin to offer sound generic diagnoses and more robust hypotheses of relationships. We encourage our colleagues to join us in this quest.

Likewise, the morphology of pyrochroid pupae is unknown except for the seven North American species briefly described by Young [19]. Our present description of the pupa for *P. reni* **n. sp.** is the first for the genus. Thus, it is impossible to provide a generic diagnosis for pupae of *Pseudopyrochroa* at this time. We are in need of many additional contributions to make this possible.

### 4.2. Molecular Phylogenetics

The research history of phylogenetics relating to Pyrochroidae was summarized by Young and Pollock [40]. Nearly all past contributions that focus on the family have been based on external gross morphological characters. Therefore, although very preliminary and incomplete, the present work is nonetheless the first molecular phylogenetic analysis of taxa within the family. The greater phylogenetic implications are nominal. However, the results clearly enabled us to associate the larva, the pupa, and the adult of *P. reni* **n. sp.**, and provided a tool to help associate larvae and pupae with known adults. Moreover, the additional *COI* barcode sequences will be useful to help clarify generic and species-level phylogenetic relationships of Pyrochroidae.

In addition, another interesting result was illustrated from the present analysis: The ML tree (Figure 7) showed that the relationship of the genera *Pseudopyrochroa*, *Schizotus*, and *Dendroides* is ((*Pseudopyrochroa* + *Schizotus*) + *Dendroides*). This relationship is somewhat different from the comparative morphological results of larvae, pupae (see above), and adults (head with cranial pits between eyes in *Pseudopyrochroa*, behind eyes in *Schizotus*, and lacking in *Dendroides*; parameres and penis with recurved apical hooks in *Pseudopyrochroa*; hooks lacking in *Schizotus* and *Dendroides*). The resolution of these incongruences will be possible only with the molecular data analysis of far more genera and species of Pyrochroinae and other subfamilies.

### 4.3. Fauna of the Family Pyrochroidae from Mt. Qinling

The Qinling Mountains-Huaihe River Line is the most consequential north-south geographical dividing line in China [41]. Meanwhile, Mt. Qinling is one of the priority areas for biodiversity conservation in China [42], and west in Gansu, through Shaanxi, east to Henan. However, with respect to Pyrochroidae, our knowledge of species richness, abundance, natural history, and phenology in this region is poor. Before the present study, only one species, *Phyllocladus kasantsevi* Young, 2005 (endemic to Gansu and Shaanxi provinces, China) was recorded [43]. *Pseudopyrochroa reni* **n. sp.** is the second recorded species of the family Pyrochroidae, and also the first species of its genus from Mt. Qinling. We also report here that the two species of *Eupyrochroa* Blair, 1914, *E. insignata* (Fairmaire, 1894) and *E. limbaticollis* (Pic, 1909), have been found from this area.

The molecular phylogenetic results revealed three species in our larval collections. Unfortunately, all adult specimens of the other pyrochroid species from Mt. Qinling are deposited as dry specimens. Attempts to extract DNA from these specimens and amplify their *COI* fragments were unsuccessful. Therefore, we could not associate the other two larval species to their respective adults. We could hypothesize that there should be another species of *Pseudopyrochroa* or *Schizotus* distributed in this area, because of the position of P1A7 in the ML tree (Figure 7). However, with no molecular sequences for *Eupyrochroa*, *Phyllocladus*, other species of *Pseudopyrochroa*, or other genera potentially in the region, it would be premature to do so at this time.

## 5. Conclusions

In the present study, the larva, pupa, and adult of a new *Pseudopyrochroa* species were described and illustrated from the western region of Mt. Qinling (Gansu, China). The three stages were associated using molecular phylogenetic analyses based on mtDNA *COI* barcode sequences. However, it remains premature to attempt a generic diagnosis for larvae and pupae of *Pseudopyrochroa*. Such an attempt must be predicated on the study and formal descriptions of larvae and pupae of many additional species.

Meanwhile, our preliminary phylogenetic analysis suggested the relationship of the genera *Pseudopyrochroa*, *Schizotus*, and *Dendroides* to be ((*Pseudopyrochroa* + *Schizotus*) + *Dendroides*). While this is somewhat different than intuitive results from comparative external morphological features, we acknowledge that our results are very preliminary. We suggest that these incongruences will be more fully resolved by phylogenetic analysis of the subfamily Pyrochroinae, including far more genera, species, and gene fragments.

Finally, the fauna of Pyrochroidae from Mt. Qinling was discussed. Four pyrochroid species are presently known to occur in this biodiversity conservation region. Our collections, including the as yet unassociated pyrochroid larvae (could be easily distinguished by the shape of urogomphal plates and urogomphi), suggest that several additional pyrochroid species will be confirmed from this area.

## Figures and Tables

**Table 1:** Species, codes, and sampling locality for the molecular analyses based on *COI* sequences.

Taxon	Stage	Sample Code	Locality/Region	GenBank Accession	Source of Data
*Pseudopyrochroa reni* Pan & Young, **n. sp.**	Adult (male)	P1B5	China, Gansu, Maiji, Liyuan Forestry Farm; 34°26.782′ N 106°31.256′ E, Elev. 1065 m	MZ902331	Current study
	Adult (female)	P1D6	China, Gansu, Maiji, Tailu Forestry Farm; 34°27.489′ N 106°13.014′ E, Elev. 1451 m	MZ902326	Current study
	Larva	P1C4 *	China, Gansu, Maiji, Liyuan Forestry Farm; 34°26.782′ N 106°31.256′ E, Elev. 1065 m	MZ902330	Current study
	Pupa	P1E4 *	China, Gansu, Maiji, Tailu Forestry Farm; 34°27.489′ N 106°13.014′ E, Elev. 1451 m	MZ902329	Current study
	Pupa	P1F1 *	China, Gansu, Maiji, Tailu Forestry Farm; 34°30.641′ N 106°13.248′ E, Elev. 1138 m	MZ902327	Current study
	Pupa	P1F4 *	China, Gansu, Maiji, Tailu Forestry Farm; 34°30.641′ N 106°13.248′ E, Elev. 1138 m	MZ902328	Current study
Pyrochroinae sp.1	Larva	P1A7 *	China, Gansu, Maiji, Longmen Forestry Farm; 34°12.607′ N 106°24.032′ E, Elev. 1347 m	MZ902332	Current study
Pyrochroinae sp.2	Larva	P1F7 *	China, Gansu, Maiji, Tailu Forestry Farm; 34°30.641′ N 106°13.248′ E, Elev. 1138 m	MZ902333	Current study
*Schizotus cervicalis* Newman, 1838	n/a	n/a	n/a	KM849000	unpublished
	n/a	n/a	n/a	MG058573	unpublished
	n/a	n/a	n/a	KM847589	unpublished
*Schizotus pectinicornis* (Linnaeus, 1758)	n/a	n/a	n/a	JF889751	unpublished
	n/a	n/a	Finland	KJ963138	[25]
	n/a	n/a	n/a	KU906128	unpublished
	n/a	n/a	n/a	KM441758	[26]
*Dendroides ephemeroides* (Mannerheim, 1852)	n/a	n/a	n/a	KM843428	unpublished
	n/a	n/a	n/a	KM845851	unpublished
*Dendroides canadensis* Latreille, 1810	n/a	n/a	Canada	KR486730	[27]
	n/a	n/a	n/a	JF887706	unpublished
*Dendroides concolor* (Newman, 1838)	n/a	n/a	n/a	JF887522	unpublished
	n/a	n/a	Canada	KR481585	[27]
	n/a	n/a	n/a	HM411878	unpublished
	n/a	n/a	n/a	HM411795	unpublished
*Pyrochroa serraticornis* (Scopoli, 1763)	n/a	n/a	n/a	JF889533	unpublished
	n/a	n/a	n/a	JF889487	unpublished
	n/a	n/a	France, Essonne, forêt domaniale de Verrières; 48.763 N 2.242 E	KM285809	[28]
*Pyrochroa coccinea* (Linnaeus, 1760)	n/a	n/a	n/a	JF889545	unpublished
	n/a	n/a	Finland	HM909042	[25]
	n/a	n/a	n/a	KU906910	unpublished
*Pedilus elegans* (Hentz, 1830)	n/a	n/a	n/a	MG057627	unpublished
	n/a	n/a	Canada	KR481560	[27]
	n/a	n/a	Canada	KR487076	[27]
*Pedilus abnormis* (Horn, 1874)	n/a	n/a	n/a	KM843886	unpublished
*Pedilus lugubris* (Say, 1827)	n/a	n/a	n/a	MG060167	unpublished
	n/a	n/a	Canada	KR481730	[27]
*Pedilus terminalis* (Say, 1827)	n/a	n/a	Canada	KR486509	[27]
	n/a	n/a	Canada	KR480871	[27]
*Boros schneideri* (Panzer, 1796)	n/a	n/a	n/a	KM444916	[26]

* Samples that were taxonomically determined following this molecular study.

**Table 2:** Maximum and mean intraspecific pairwise divergence of 14 pyrochroid species.

Species	Number of Samples	Intraspecific Distance (%)	
		Maximum	Mean
*Pseudopyrochroa reni* **n. sp.**	6	2.17	1.30
Pyrochroinae sp.1	1	n/a	n/a
Pyrochroinae sp.2	1	n/a	n/a
*Schizotus cervicalis*	3	0.92	0.82
*Schizotus pectinicornis*	4	1.23	0.69
*Dendroides ephemeroides*	2	0.92	0.92
*Dendroides canadensis*	2	0.31	0.31
*Dendroides concolor*	4	2.33	1.39
*Pyrochroa serraticornis*	3	4.41	2.94
*Pyrochroa coccinea*	3	0.46	0.31
*Pedilus elegans*	3	0.15	0.10
*Pedilus abnormis*	1	n/a	n/a
*Pedilus lugubris*	2	0.00	0.00
*Pedilus terminalis*	2	0.15	0.15

## Data Availability

The data of the research were deposited at College of Life Sciences, Hebei University, Baoding, China and Department of Entomology, University of Wisconsin, Madison, U.S.A. The specimens associated with this paper, as well as the sequence data submitted to GenBank, conform to the 2014 Nagoya Protocol on Access to Genetic Resources and the Fair and Equitable Sharing of Benefits Arising from their Utilization (https://www.cbd.int/abs/, accessed on 8 November 2021).

## References

[1] Young D.K. (1999). Transfer of the Taiwanese *Pseudopyrochroa umenoi* and the Japanese *P. amamiana* to *Pseudodendroides* (Coleoptera: Pyrochroidae: Pyrochroinae). Pan-Pac. Entomol..

[2] Kai T., Yoshitomi H. (2018). A revision of *Pseudopyrochroa* (Coleoptera: Pyrochroidae: Pyrochroinae) from Japan. Jpn. J. Syst. Entomol..

[3] Young D.K., Telnov D., Pollock D., Iwan D., Löbl I. (2020). Family Pyrochroidae Latreille, 1806. Catalogue of Palaearctic Coleoptera, Volume 5: Tenebrionoidea.

[4] Blair K.G. (1914). A revision of the family Pyrochroidae (Coleoptera). Ann. Mag. Nat. Hist..

[5] Kôno H. (1929). Die Pyrochroiden Japans. Insecta Matsumurana.

[6] Pic M. (1906). Contribution à l’étude des pyrochroides. L’Échange Rev. Linnéenne.

[7] Young D.K. (2000). Five new species of *Pseudopyrochroa* (Coleoptera: Pyrochroidae: Pyrochroinae) from Taiwan. Orient. Insects.

[8] Young D.K. (2019). A new *Pseudopyrochroa* Pic, 1906 from Yunnan, China with a key to adult *Pseudopyrochroa* males from the Province and correction on type repository for *Frontodendroidopsis pennyi* Young (Coleoptera: Pyrochroidae: Pyrochroinae). Zootaxa.

[9] Young D.K. (2001). A new species of *Pseudopyrochroa* (Coleoptera: Pyrochroidae: Pyrochroinae) from China. Orient. Insects.

[10] Young D.K. (2003). A new species of *Pseudopyrochroa* (Coleoptera, Pyrochroidae, Pyrochroinae) from China and Korea. Spec. Bull. Jpn. Soc. Coleopterol. Tokyo.

[11] Pic M. (1908). Deux nouveaux *Pyrochroa* Geoffr. de Chine [Col.]. Bull. De La Société Entomol. De Fr..

[12] Pic M. (1911). Coléoptères exotiques nouveaux ou peu connus (Suite). L’Échange Rev. Linnéenne.

[13] Pic M. (1914). Descriptions abrégées de malacodermes et hétéromères. Mélanges Exot.-Entomol..

[14] Pic M. (1939). Deux nouveaux coléoptères de Chine. Notes D’entomologie Chin. Musée Heude.

[15] Pic M. (1955). Coléoptères nouveaux de Chine. Bull. De La Société Entomol. De Mulhouse.

[16] Young D.K., Hsiao Y., Liang W.-R., Lee C.-F. (2016). Description of the mature larvae for two species of *Pseudopyrochroa* from Taiwan (Coleoptera: Pyrochroidae: Pyrochroinae), with notes on their natural history. Zootaxa.

[17] Young D.K., Hsiao Y. (2016). Description of the mature larva of *Pseudopyrochroa depressa* (Pic) (Coleoptera: Pyrochroidae: Pyrochroinae), with comparison to other Taiwanese *Pseudopyrochroa*. Zootaxa.

[18] Yoshitomi H., Kai T. (2015). Larvae and check list of Japanese Pyrochroidae. Sayabane.

[19] Young D.K. (1975). A revision of the family Pyrochroidae (Coleoptera: Heteromera) for North America based on the larvae, pupae, and adults. Contrib. Am. Entomol. Inst..

[20] Pollock D.A. (1994). Systematic position of Pilipalpinae (Coleoptera: Tenebrionoidea) and composition of Pyrochroidae. Can. Entomol..

[21] Beutel R.G., Friedrich F. (2005). Comparative study of larvae of Tenebrionoidea (Coleoptera: Cucujiformia). Eur. J. Entomol..

[22] Kergoat G.J., Soldati L., Clamens A.-L., Jourdan H., Jabbour-Zahab R., Genson G., Bouchard P., Condamine F.L. (2014). Higher level molecular phylogeny of darkling beetles (Coleoptera: Tenebrionidae). Syst. Entomol..

[23] McKenna D.D., Wild A.L., Kanda K., Bellamy C.L., Beutel R.G., Caterino M.S., Farnum C.W., Hawks D.C., Ivie M.A., Jameson M.L. (2015). The beetle tree of life reveals that Coleoptera survived end-Permian mass extinction to diversify during the Cretaceous terrestrial revolution. Syst. Entomol..

[24] McKenna D.D., Shin S., Ahrens D., Balke M., Beza-Beza C., Clarke D.J. (2019). The evolution and genomic basis of beetle diversity. Proc. Natl. Acad. Sci. USA.

[25] Pentinsaari M., Hebert P.D.N., Mutanen M. (2014). Barcoding beetles: A regional survey of 1872 species reveals high identification success and unusually deep interspecific divergences. PLoS ONE.

[26] Hendrich L., Morinière J., Haszprunar G., Hebert P.D.N., Hausmann A., Köhler F., Balke M. (2015). A comprehensive DNA barcode database for Central European beetles with a focus on Germany: Adding more than 3500 identified species to BOLD. Mol. Ecol. Resour..

[27] Hebert P.D.N., Ratnasingham S., Zakharov E.V., Telfer A.C., Levesque-Beaudin V., Milton M.A., Pedersen S., Jannetta P., Dewaard J.R. (2016). Counting animal species with DNA barcodes: Canadian insects. Philos. Transctions R. Soc. B Biol. Sci..

[28] Rougerie R., Lopez-Vaamonde C., Barnouin T., Delnatte J., Moulin N., Noblecourt T., Nusillard B., Parmain G., Soldati F., Bouget C. (2015). PASSIFOR: A reference library of DNA barcodes for French saproxylic beetles (Insecta, Coleoptera). Biodivers. Data J..

[29] Folmer O., Black M., Hoeh W., Lutz R., Vrijenhoek R. (1994). DNA primers for amplification of mitochondrial cytochrome c oxidase subunit I from diverse metazoan invertebrates. Mol. Mar. Biol. Biotechnol..

[30] Liu S.-P., Pan Z., Ren G.-D. (2016). Identification of three morphologically indistinguishable *Epicauta* species (Coleoptera, Meloidae, Epicautini) through DNA barcodes and morphological comparisons. Zootaxa.

[31] Katoh K., Rozewichi J., Yamada K.D. (2019). MAFFT online service: Multiple sequence alignment, interactice sequence choice and visualization. Brief. Bioinform..

[32] Kumar S., Stecher G., Li M., Knyaz C., Tamura K. (2018). MEGA X: Molecular Evolutionary Genetics Analysis across computing platforms. Mol. Biol. Evol..

[33] Felsenstein J. (1981). Evolutionary trees from DNA sequences: A maximum likelihood approach. J. Mol. Evol..

[34] Kalyaanamoorthy S., Minh B.Q., Wong T.K.F., von Haeseler A., Jermiin L.S. (2017). ModelFinder: Fast model selection for accurate phylogenetic estimates. Nat. Methods.

[35] Zhang D., Gao F., Jakovlić I., Zou H., Zhang J., Li W.X., Wang G.T. (2020). PhyloSuite: An integrated and scalable desktop platform for streamlined molecular sequence data management and evolutionary phylogenetics studies. Mol. Ecol. Resour..

[36] Nguyen L.T., Schmidt H.A., von Haeseler A., Minh B.Q. (2015). IQ-TREE: A fast and effective stochastic algorithm for estimating maximum-likelihood phylogenies. Mol. Biol. Evol..

[37] Hayashi N. (1969). On the larvae of Pyrochroidae occurring in Japan. Kontyû.

[38] Minh B.Q., Nguyen M.A.T., von Haeseler A. (2013). Ultrafast approximation for phylogenetic bootstrap. Mol. Biol. Evol..

[39] Guindon S., Dufayard J.-F., Lefort V.L., Anisimova M., Hordijk W., Gascuel O. (2010). New algorithms and methods to estimate maximum-likelihood phylogenies: Assessing the performance of PhyML 3.0. Syst. Biol..

[40] Young D.K., Pollock D.A., Beutel R.G., Lawrence J.F. (2010). Pyrochroidae Latreille, 1807. Handbook of Zoology. Coleoptera, Beetles, Volume 2: Morphology and Systematics (Elateroidea, Bostrichiformia, Cucujiformia partim).

[41] Zhang J., Liu X.-N., Tan Z.-H., Chen Q.-G. (2012). Mapping of the north-south demarcation zone in China based on GIS. J. Lanzhou Univ..

[42] Ministry of Ecology and Environment of the People’s Republic of China Notice on issuing the Scope of Priority Areas for Biodiversity Conservation in China. http://www.mee.gov.cn/gkml/hbb/bgg/201601/t20160105_321061.htm.

[43] Young D.K. (2005). A new species of *Phyllocladus* (Coleoptera: Pyrochroidae: Pyrochroinae) from China, with a key to males of the two known Chinese species. Orient. Insects.

